# Different COVID-19 treatments’ impact on hospital length of stay

**DOI:** 10.1186/s40001-023-01201-8

**Published:** 2023-07-03

**Authors:** Satori Iwamoto, Bahaar Kaur Muhar, Hao Chen, Harrison Chu, Mason Johnstone, Ashwin Sidhu, Hillary Chu, Joseph Fischer, Gary Chu

**Affiliations:** 1 California Northstate University College of Medicine, Elk Grove, USA; 2 Department of Respiratory Internal Medicine, Yokohama City University, Yokohama, Japan

**Keywords:** COVID-19, Length of stay, Mortality

## Abstract

**Importance:**

COVID-19 has adversely affected global healthcare infrastructure since 2019. Currently, there are no large-scale published reports on the efficacy of combination therapy of dexamethasone, remdesivir, and tocilizumab on COVID-19 patients.

**Objectives:**

Is the combination therapy of dexamethasone, remdesivir, and tocilizumab superior to other treatments on hospitalized COVID-19 patients?

**Design:**

This is a retrospective, comparative effectiveness study.

**Setting:**

Single-center study

**Participants/interventions:**

We analyzed different inpatient COVID-19 treatment options available in the United States and their impact on hospital length of stay (LOS) and mortality. Hospitalized COVID-19 were categorized as “mild,” “moderate” and “severe'' based on the highest level of oxygen required; room air, nasal cannula, or high flow/PAP/intubation, respectively. Patients were treated in accordance with the availability of medications and the latest treatment guidelines.

**Main outcomes:**

The endpoints of the study are hospital discharges and death during hospitalization.

**Results:**

1233 COVID-19 patients were admitted from 2020 to 2021. No treatment combinations showed a statistically significant decrease in hospital LOS in mild COVID-19 patients (*p* = 0.186). In moderate patients, the combination of remdesivir and dexamethasone slightly decreased LOS by 1 day (*p* = 0.007). In severe patients, the three-drug combination of remdesivir, dexamethasone, and tocilizumab decreased LOS by 8 days (*p* = 0.0034) when compared to nonviable treatments, such as hydroxychloroquine and convalescent plasma transfusion. However, it did not show any statistically significant benefit when compared to two-drug regimens (dexamethasone plus remdesivir) in severe COVID-19 (*p* = 0.116). No treatment arm appeared to show a statistically significant decrease in mortality for severe COVID-19 patients.

**Conclusions:**

Our findings suggest that three-drug combination may decrease LOS in severe COVID-19 patients when compared to two-drug therapy. However, the trend was not supported by statistical analysis. Remdesivir may not be clinically beneficial for mild hospitalized COVID-19 patients; considering its cost, one could reserve it for moderate and severe patients. Triple drug therapies, while potentially reducing LOS for severe patients, do not affect overall mortality. Additional patient data may increase statistical power and solidify these findings.

## Key points

- Question: Is the three-drug combination therapy of dexamethasone, remdesivir, and tocilizumab effective in decreasing the length of stay for patients with severe COVID-19 disease?.
- Findings: The three-drug combination of remdesivir, dexamethasone, and tocilizumab decreased LOS by 8 days (p=0.0034) compared to nonviable treatments like hydroxychloroquine and convalescent plasma transfusion. However, it did not show any statistically significant benefit when compared to two-drug regimens (dexamethasone plus remdesivir) in severe COVID-19 (p=0.116). No treatment arm appeared to show a statistically significant decrease in mortality for severe COVID-19 patients.
- Meaning: Triple drug therapies that include tocilizumab may potentially reduce LOS for severe patients but we did not find statistically significant data to support this statement

## Introduction

The highly contagious SARS-CoV-2 causes the Coronavirus disease (COVID-19), first discovered in Wuhan, China, in December 2019 [1]. The COVID-19 pandemic has adversely affected world economies, global public health infrastructure, and social behaviors. Despite physical distancing and preventative precautions, such as face masks, tens of thousands of people are still being affected by COVID-19 [2]. While it is a disease that mainly involves the lungs, multiple organs, including neurological symptoms, have been described frequently [3]. Even young adults with seemingly asymptomatic COVID-19 infections can end up suffering a thrombotic stroke that adversely affects their health [4]. Currently, there is no definitive antiviral agent to treat hospitalized patients with severe COVID-19 [5]. However, any treatment that may decrease hospital length of stay will still be beneficial, especially during the height of the pandemic when many hospitals are near or at full capacity [6]. Fortunately, combination therapies with pre-existing agents have shown promising clinical improvement and repurposing FDA-approved drugs may prove beneficial. Current SARS-CoV-2 drug therapies may be divided into those that (1) target the RNA or proteins of the virus and (2) biologics that interact and interfere with host proteins and processes supporting the virus. From March 2020 to October 2021, the recommendation was to use dexamethasone and tocilizumab for hospitalized COVID-19 patients requiring mechanical ventilation and dexamethasone and remdesivir for hospitalized COVID-19 patients not experiencing hypoxia or requiring supplemental oxygen without mechanical ventilation [7, 8].

Dexamethasone is a ubiquitous corticosteroid that is widely available at low cost [9]. It was the first major “breakthrough” therapy in treating COVID-19 and has been shown by the RECOVERY trials to increase the number of ventilator-free days and reduce the risk of death [8, 10]. Remdesivir is an antiviral drug, given emergency use authorization for SARS-CoV-2 in 2020, that was originally used in clinical trials against Ebola [10]. It is currently used to treat hospitalized COVID-19 patients not experiencing hypoxia or those requiring supplemental oxygen without mechanical ventilation. Remdesivir has previously been shown to reduce early stage mortality and the need for high-flow oxygen supplementation and invasive mechanical ventilation amongst hospitalized COVID-19 patients—with an increased clinical recovery rate of 21% on day 7 and 29% on day 14 of treatment compared to the placebo group [11]. Originally approved for rheumatoid arthritis, tocilizumab is also used for COVID-19. Tocilizumab is a recombinant humanized IgG1 monoclonal antibody that reduces the pro-inflammatory effect of IL-6 and has previously been shown to reduce mortality and the need for mechanical ventilation in COVID-19 patients [12, 13]. Prior reports analyzing trials of combination treatments for COVID-19 have had mixed results [14]. Other treatments such as hydroxychloroquine, chloroquine, convalescent plasma transfusions, ivermectin, and combination therapy with protease inhibitors such as Lopinavir + Ritonavir and Darunavir + Cobicistat either had no change or increase in mortality [15, 16].

Different treatments, alone or in combination, not only impacts patient morbidity and outcomes but also hospital LOS [17]. During the peak phase of the pandemic, hospitals were inundated with critical patients requiring inpatient hospital admission resulting in hospital staff, bed, and supply shortages and an increasing number of patients awaiting admission despite hospitals operating at maximum capacity [18]. Therefore, assessing the LOS of admitted COVID-19 patients of varying severity based on different treatment arms would be invaluable in assisting resource management of hospital staff and oxygen supplies [6].

## Methods

In this retrospective cohort study, we investigated adults (18 +) hospitalized with COVID-19 at a community hospital in northern California from March 2020 to March 2021 and September 2021 to October 2021 to evaluate if the combination treatment of (1) remdesivir + dexamethasone, or (2) remdesivir + dexamethasone + tocilizumab truly decreased hospital LOS for non-hypoxic and hypoxic patients as well as its effect on 30-day mortality. In addition, we include data from treatments such as hydroxychloroquine and convalescent plasma transfusion for the sake of inclusion and academic discussion, and because a significant segment of American society, is still firmly entrenched in various conspiracy theories and medications that have no known benefit to the treatment of COVID-19[19].

### Inclusion and exclusion criteria

The following demographics are excluded: pediatric patients, patients hospitalized for primarily non-COVID-19 reasons (i.e., upper gastrointestinal bleeding or pregnancy), and patients on comfort care. Importantly, patients with LOS 30 days were excluded from the study as well as they usually have other medical and/or social variables affecting LOS [20].

### Definition

COVID-19 patients were categorized into different categories: “mild,” “moderate” and “severe” based on the highest level of oxygen needs. Mild COVID-19 cases refer to patients in room air (RA) during their entire hospitalization. Moderate patients use oxygen supplements via nasal cannula (NC) during their hospital stay. Severe COVID-19 patients require the support of one or more of the following oxygen delivery devices: high flow oxygen (HFNC), continuous positive airway pressure (CPAP) machine, bilevel positive airway pressure (BiPAP) machine, or intubation with mechanical ventilation.

### Outcomes

The endpoints of the study include the date of discharge and death during hospitalization. Medical records were analyzed and the following data were extracted: patient’s age, gender, oxygen supplementation usage, COVID-19 treatment, and LOS. COVID-19 treatment included dexamethasone alone, remdesivir alone, dexamethasone + remdesivir, dexamethasone + remdesivir + tocilizumab, “other” and none. “Other” treatments included giving only hydroxychloroquine, only convalescent plasma, dexamethasone + remdesivir + convalescent plasma therapy, and dexamethasone + convalescent plasma therapy.

### Data analysis

JMP Pro Statistical Software was used for all statistical analysis and development of charts (mosaic and diamond plots) and tables. We used the one-way ANOVA test to see if our findings were statistically significant. Our statistical analysis included regression analysis, mean, and 95% confidence interval (CI). In addition, to ensure the accuracy of the diagnostic codes being extracted, 100% of the codes were verified by the Principal Investigator.

## Results

1233 COVID-19 patients were hospitalized in Kaiser South Sacramento from March 2020 to March 2021 and September 2021 to October 2021. 53.4% of these patients were male and 46.6% were female but there was no statistically significant difference in the ratio of gender (F:M) in each treatment type (*p* = 0.080). Similarly, the distribution of ages between different treatment groups was indistinguishable and, therefore, insignificant (*p* = 0.002).

A total of 1233 patients were studied. 233 patients were excluded by the criteria listed above. Of the 1000 patients analyzed, 109 were “mild”, 510 were “moderate” and 381 were “severe” COVID-19 patients. There was a statistically significant difference between the severity of COVID-19 patients and the treatment type (*p* < 0.001).

Most of these patients, 665 (62.9%), were treated with a combination of dexamethasone and remdesivir. 216 (20.4%) were treated with dexamethasone only. 67 (6.3%) were given remdesivir only. 47 (4.4%) received the combination therapy of dexamethasone, remdesivir, and tocilizumab. 33 patients received remdesivir, dexamethasone, and baricitinib combination therapy (3.1%). A small subset, 30 (2.8%), were treated with “other” methods (see methods section).

**Fig. 1:**
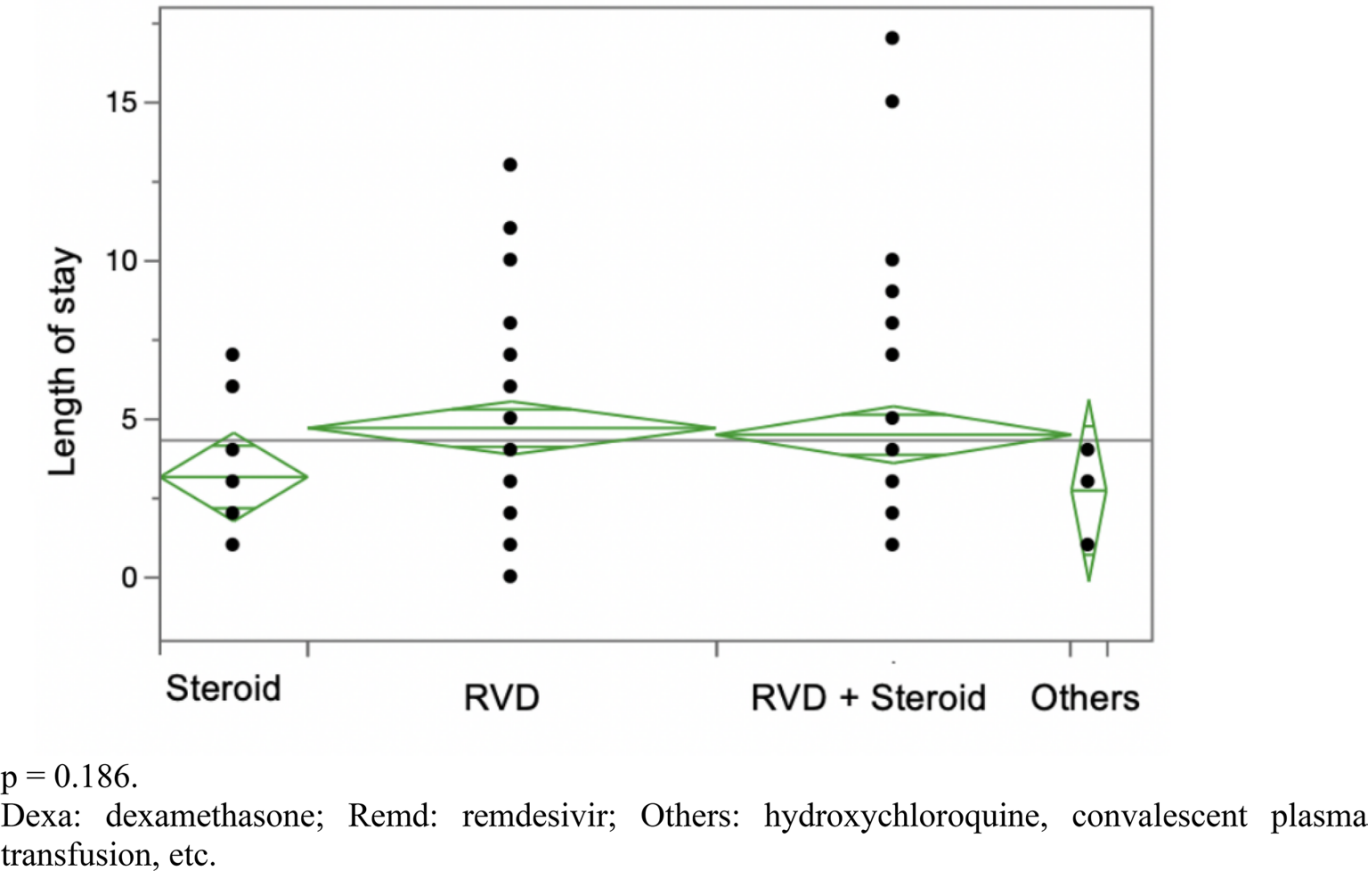
Hospitalized patients with mild COVID-19 *p* = 0.186. *Dexa* dexamethasone, *Remd* remdesivir, *Others* hydroxychloroquine, convalescent plasma transfusion, etc.

**Fig. 2:**
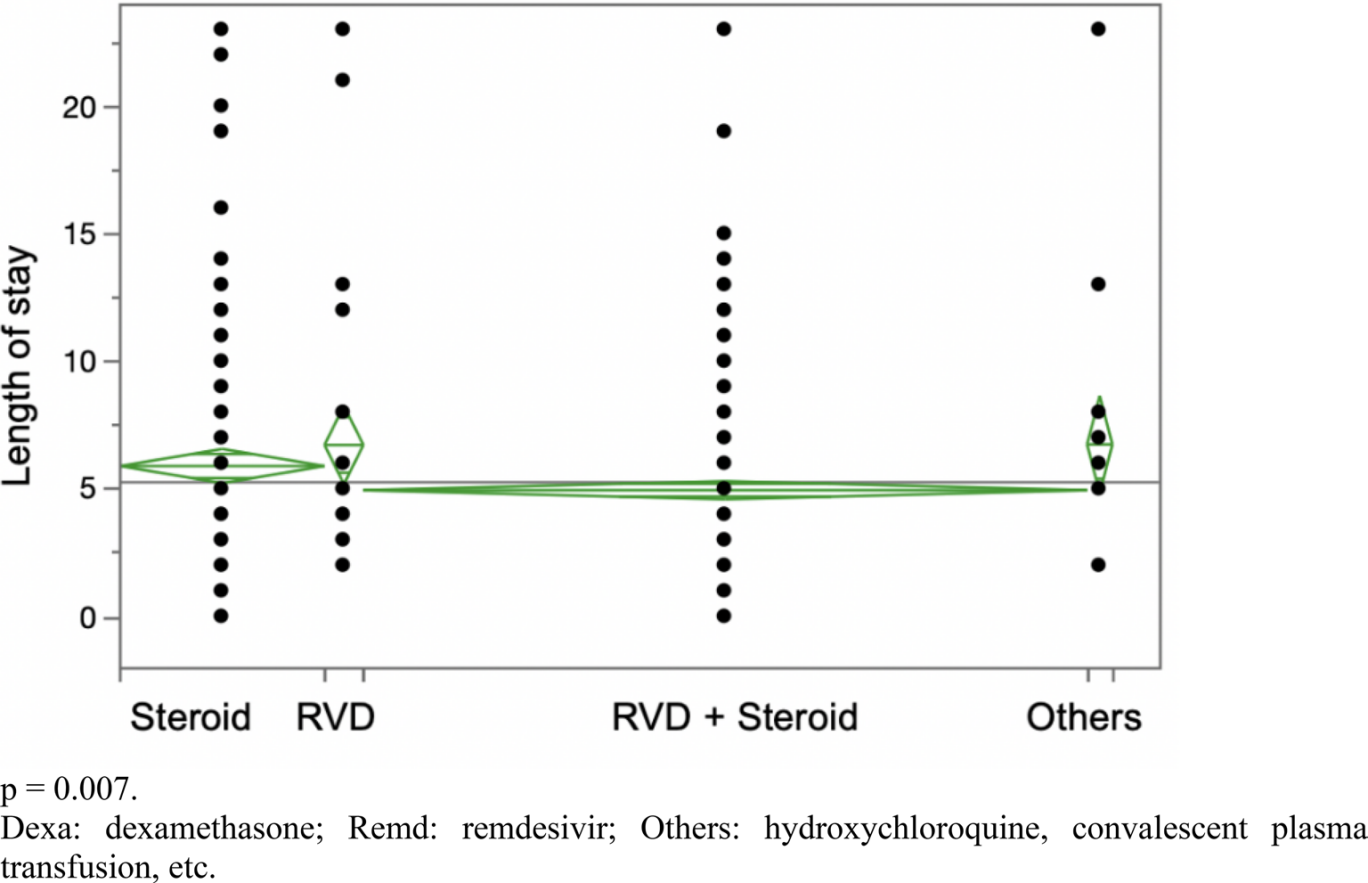
Hospitalized patients with moderate COVID-19. *p* = 0.007. *Dexa* dexamethasone, *Remd* remdesivir, *Others* hydroxychloroquine, convalescent plasma transfusion, etc.

**Fig. 3:**
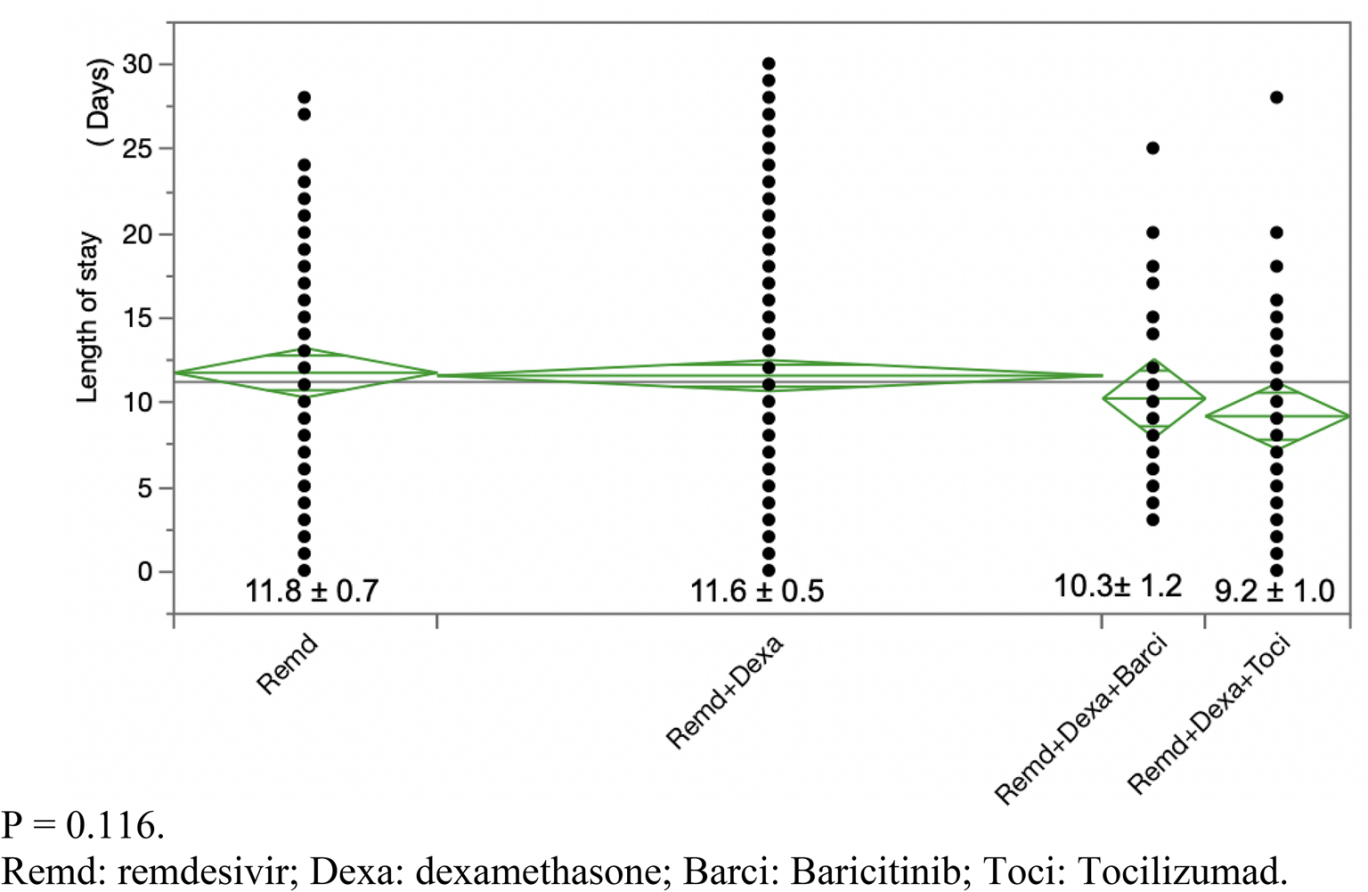
Hospitalized patients with severe COVID-19. *P* = 0.116. *Remd* remdesivir, *Dexa* dexamethasone, *Barci* Baricitinib, *Toci* Tocilizumad

**Fig. 4:**
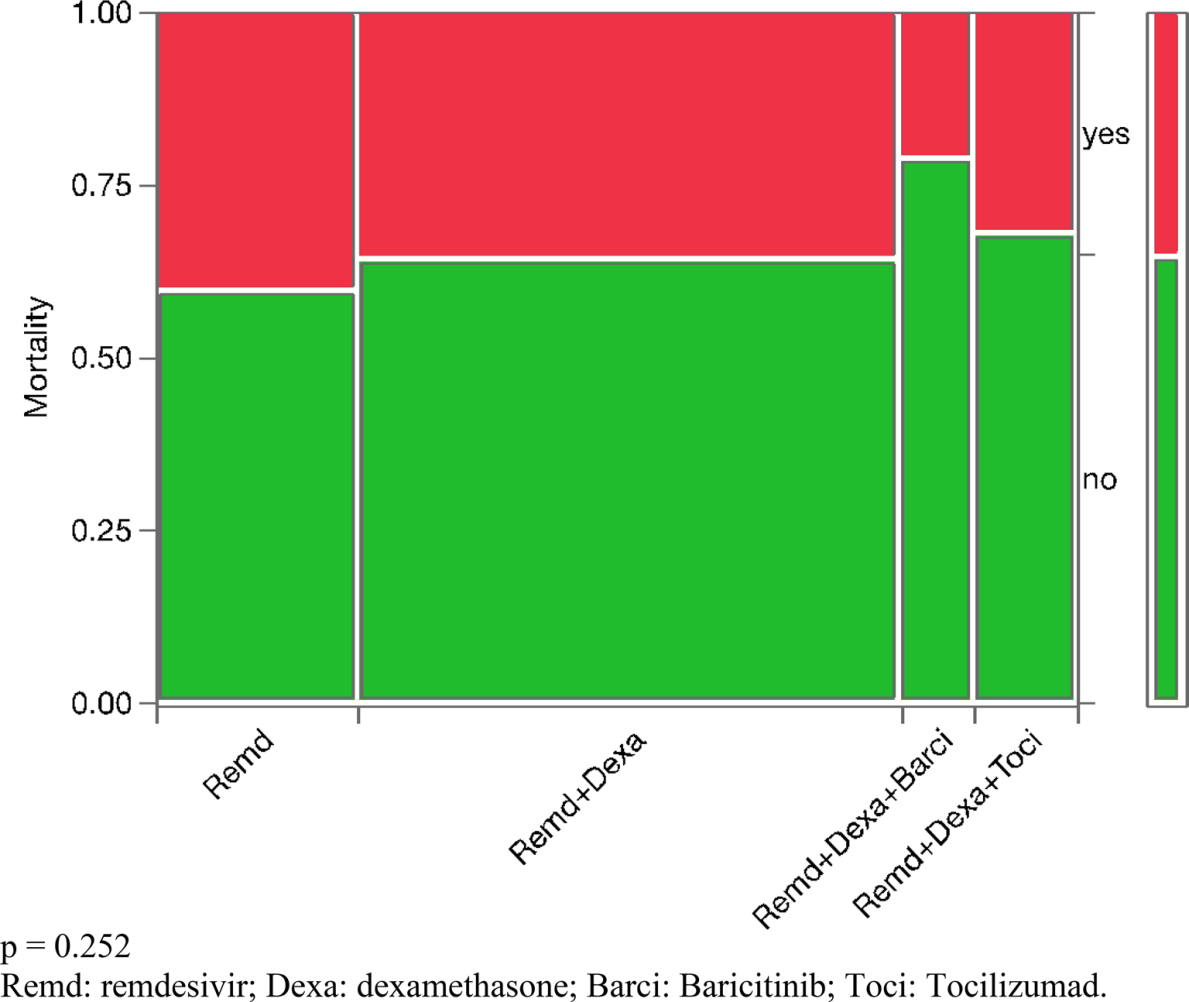
Mortality in hospitalized patients with severe COVID-19. *p* = 0.252. *Remd* remdesivir, Dexa: dexamethasone, *Barci* Baricitinib, *Toci* Tocilizumad

In mild COVID-19 patients, the LOS did not show any statistical difference between different treatment arms with a *p* value of 0.186 (Fig. 1, Table 1). In moderate COVID-19 patients, the combination treatment of dexamethasone plus remdesivir showed a statistically significant reduction in LOS from 6.77 days to 4.98 days with a *p* value of 0.007 (Fig. 2, Table 1). In severe COVID-19 patients, the combination treatment of remdesivir, dexamethasone, and tocilizumab showed a statistically significant reduction in LOS of up to 8 days (*p* = 0.0034) when compared to “other,'' nonviable treatments, such as hydroxychloroquine or convalescent plasma transfusion (Table 1). However, upon excluding the “other” category, there was no statistically significant difference between various combination treatments with a *p* value of 0.116 (Fig. 3). Overall, there was no significant reduction in mortality for severe COVID-19 patients between different combination treatments with a *p* value of 0.252 (Fig. 4).

**Table 1:** LOS = length of stay. RMD = remdesivir

LOS In Mild COVID-19 Patients			
Treatment type	Number (n)	Mean	Standard deviation
Remdesivir	47	4.723	0.423
Remdesivir + dexamethasone	41	4.512	0.4529
Dexamethasone	17	3.176	0.4529
Others (Hydroxychloroquine/Transfusion/ETC)	4	2.75	1.45
*p* = 0.186			
LOS In Moderate COVID-19 Patients			
Dexamethasone	105	5.924	0.341
Remdesivir	20	6.75	0.782
Remdesivir + dexamethasone	372	4.978	0.181
Others (Hydroxychloroquine/Transfusion/ETC)	13	6.377	4.864
*p* = 0.007			
LOS In Severe COVID-19 Patients			
Dexamethasone	82	11.768	0.75
Dexa + RMD	211	11.578	0.46
Dexa + RMD + Baricitinib	32	10.25	1.2
Dexa + RMD + Tocilizumab	45	9.2	1.01
Others (Hydroxychloroquine/Transfusion/ETC)	11	17.909	2.05
*p* = 0.0034			

Mild patients are not hypoxic and on room-air only. Moderate patients require nasal cannula for oxygen support. Severe patients need high flow oxygen nasal cannula, PAP, or intubation with mechanical ventilation support to keep oxygen saturation greater than 90%

## Discussion

Prior studies have indicated that the combination of dexamethasone and remdesivir has modest effects in reducing hospital LOS in moderate-to-severe COVID-19 patients [21]. In our study, only moderate patients (NC only) given dexamethasone and remdesivir were found to have a statistically significant decreased LOS by approximately 1.5 days (Fig. 2).

During this investigation (12/31/2022), there were no conclusive published reports on the efficacy of the three-drug combination therapy of dexamethasone, remdesivir, and tocilizumab on COVID-19 patients [8]. In severe COVID-19 cases, we initially found the three-drug combination treatment (dexamethasone + remdesivir + tocilizumab) to be statistically superior to all other treatments when considering nonviable treatments, such as hydroxychloroquine or convalescent plasma transfusion. (Table 1) This is in accordance with the current literature [22]. However, the statistical power of this finding, a *p* value of 0.0034, came mostly from the grossly poor LOS associated with the nonviable treatment group. When we removed nonviable treatment data from the analysis, three-drug combination treatments failed to yield a statistically significant decrease in LOS, based on a *p* value of 0.116 (Fig. 3). We know from other trials that the clinical benefit of dexamethasone plus tocilizumab, while significant is small [8]. It is possible that the three-drug combination (dexamethasone + remdesivir + tocilizumab) offers similar minute benefits that are undetectable by our relatively small sample size. More patient data may yield statistically significant findings.

In addition, there was also no statistically significant difference between the three-drug combination of the tocilizumab group and the baricitinib group (Fig. 3). Perhaps, the high *p* value is due to the relatively small sample size (32) of the baricitinib group. Additional data samples may improve the *p* value. Finally, It should be noted that prior literature has suggested that baricitinib may not decrease LOS [23].

Current treatment guidelines, per RECOVERY and other trials, recommend dexamethasone only for hypoxic COVID-19 patients [14, 24, 25]. This is in alignment with our study as dexamethasone—individually or in combination—has no statistically significant impact on hospital LOS for mild patients on RA (Fig. 1). In addition, the remdesivir group’s LOS also shows no statistical reduction in LOS for mild COVID-19 patients (Fig. 1). Perhaps, patients with mild COVID-19 experienced minimal benefit from Remdesivir.

In our study, 47 mild patients were treated with only remdesivir, and 41 mild patients were treated with remdesivir and dexamethasone during their stay. Typically, these patients would receive five doses of remdesivir at $520 per dose, costing the healthcare facility at least $228,800 [26]. This figure does not factor in the logistic cost of administering medications (nursing and pharmacy staff). In addition, patients with rheumatoid arthritis faced drug shortage challenges—including remdesivir and steroids—during the pandemic [27]. In addition to mild patients not benefiting from remdesivir in our study, this financial relevance further suggests against the use of remdesivir on mild COVID-19 patients.

In our study, we found no mortality benefit in any treatment arm in mild, moderate, and severe COVID-19 patients (Fig. 4). These results are inconsistent with prior studies that have suggested some survival benefits for dexamethasone, remdesivir, and tocilizumab [10–12]. However, these survival benefits, while statistically significant, are often minute. Given our relatively small sample size, it is plausible that our study does not detect such survival benefits. In addition, current treatment guidelines from the ACTT-1 trial show that remdesivir only decreases hospital LOS and not mortality [28].

A study previously suggested that men may benefit more than women with 5-day treatment with remdesivir [20]. Another study reported that the male gender is overrepresented in COVID-19 treatment clinical studies [29]. Interestingly, we also have slightly more men in our database than women. Given that there are more women than men in the human population, it is perplexing to see more men hospitalized with COVID-19 than women. Perhaps, there is a behavior or genetic difference that predisposes men to catch COVID-19 more readily than women [30]. Literature on gender analysis with COVID-19 treatment types and hospital LOS is limited and, therefore, should be investigated in future studies.

The treatment of the COVID-19 geriatric population (65 >) is an important subsample to analyze as this population is the greatest to be hospitalized throughout the pandemic, with 189,735 of 418,804 cases nationally [31]. Our study had an average age of 61 in 1233 patients. There is currently not much data on age correlation with COVID-19 treatment efficacy, side effects, or hospital LOS. A recent literature review noted remdesivir and dexamethasone to have inefficiency in geriatric patients, while tocilizumab findings were inconclusive [32]. With enough sampling data, we may be able to further analyze LOS between treatments based on different age groups and the severity of the disease.

Please note, in our hospital experience, patients who did not tolerate non-invasive ventilation (NIV) were intubated. It is a recognized phenomenon that certain populations cannot tolerate NIV [33]. In addition, within the past 2 years, we have seen acute ischemic stroke as a complication of patients with severe SARS-CoV-2 infection due to its precipitation of a hypercoagulable state [34]. There has even been a reported case of stroke in a young healthy woman with an asymptomatic SARS-CoV-2 infection as well [35]. Studies have shown that in-hospital treatment with heparin was associated with lower mortality in severe COVID-19 patients [36]. All COVID-19 patients in this data were given heparin DVT prophylactic doses unless there were contraindications (i.e., bleeding).

Although our sample size and data represent statistical significance, it has limitations. First, the data collected are partial and not continuous; our study is missing patient data from April to July 2021, which includes an additional 1000 data points. We collected and included the patient population from August to September 2021 in our data analysis to observe the preliminary results on the effect of dexamethasone + remdesivir + tocilizumab treatment, as the use of tocilizumab (IL-6 inhibitor) was not granted emergency use authorization (EUA) for COVID-19 treatment in patients under supplemental oxygen or ventilation until June 24, 2021 [37]. In addition, the sample size for this combination treatment is relatively small at 45 patients.

Second, our selection criteria do not exclude or account for specific comorbidities. We found that many patients admitted as “severe” COVID-19 patients to have preexisting comorbidities, such as chronic kidney disease, liver failure, heart disease, etc. There are also many young healthy patients without comorbidities that ended up on the ventilator with severe hypoxic resp failure. Either way, those with severe resp illness received the same medication available during that given period, regardless of comorbidities. Future studies may further delineate treatment outcomes based on comorbidities.

Biomarkers that are associated with predicting clinical progression and outcome in COVID-19, such as D-dimers, CRP, and IL-6, were not included in the study. It is well-known that the level of IL-6 could be used as a predictor of the efficacy of tocilizumab [39]. However, these labs were not ordered consistently on every patient because of the variation in practices of physicians in the hospital and ongoing changes in treatment protocol throughout the pandemic [40].

Drug shortages (i.e., remdesivir) during the pandemic affected treatment options for our patients [41]. This in combination with continuous changes in national treatment protocol over the LOS may also have affected our data.

Finally, the evolution of COVID-19 variants during the pandemic is not taken into account. Viral genome sequencing was not available for every infected patient who was hospitalized, making it difficult to accurately associate the severity of the disease with certain variants. Our data analyzes patients during the three notable COVID-19 variants: alpha, beta, and delta [42]. The variants trended towards an increase in transmissibility and decrease in virulence, indicating that newer SARS-CoV-2 variants may be less virulent [43]. The emergence of vaccines during the pandemic most definitely affected the demographics of hospitalized COVID-19 patients and probably altered the LOS as well [44].

## Conclusion

The current COVID-19 regimen of dexamethasone + remdesivir in moderate COVID-19 patients decreases the hospital LOS. Combination therapy of dexamethasone + remdesivir + tocilizumab in severe COVID-19 patients may decrease the length of a patient’s hospital stay. It is unclear if three-drug combination therapies are superior to two-drug ones in severe COVID-19 patients. No treatment modality seems to offer clinical benefit for hospitalized patients with mild COVID-19 in terms of reduction in LOS or mortality. Future studies could investigate patient age, the impact of COVID-19 vaccination status, and any other risk factors.

## Data Availability

Access to the original data may be requested by Dr. Gary Chu via email—Gary.Chu@CNSU.edu.

## References

[1] Muhar BK, Nehira J, Malhotra A, Kotchoni SO (2022). The race for COVID-19 vaccines: the various types and their strengths and weaknesses. J Pharm Pract.

[2] World Health Organization, WHO coronavirus (COVID-19) dashboard., https://covid19.who.int Accessed from 15 Jan 2023.

[3] Deana C, Verriello L, Pauletto G, Corradi F, Forfori F, Cammarota G, Bignami E, Vetrugno L, Bove T (2021). Insights into neurological dysfunction of critically ill COVID-19 patients. Trends Anaesthesia Critical Care.

[4] Iwamoto S, Johnstone M, Chiu M, Chu H (2022). Acute ischemic stroke in a young woman with an otherwise asymptomatic SARS-CoV-2 infection. J Med Res Surg.

[5] Şimşek-Yavuz S, Komsuoğlu Çelikyurt FI (2021). An update of anti-viral treatment of COVID-19. Turk J Med Sci.

[6] Iwamoto S, Muhar BK, Sidhu A, Chu H, Chiu M, Spantzel H, Chu H, Zhou N, Spantzel A, Zhang E, Chu G (2022). Predicting oxygen utilization & nurse staffing needs for SARS-CoV-2. J Bioinform Neurosci.

[7] U.S. Department of Health and Human Services, Hospitalized adults: Therapeutic management. National Institutes of Health., https://www.covid19treatmentguidelines.nih.gov/tables/therapeutic-management-of-hospitalized-adults/ Accessed from 15 Jan 2023.

[8] Horby P, Lim WS, Emberson JR, Mafham M, Bell JL, Linsell L, Staplin N, Brightling C, Ustianowski A, Elmahi E, Prudon B, Green C, Felton T, Chadwick D, Rege K, Fegan C, Chappell LC, Faust SN, Jaki T, Jeffery K, Montgomery A, Rowan K, Juszczak E, Baillie JK, Haynes R, Landray MJ (2021). Dexamethasone in hospitalized patients with Covid-19. N Engl J Med.

[9] Wolfe CR, Tomashek KM, Patterson TF, Gomez CA, Marconi VC, Jain MK, Yang OO, Paules CI, Palacios GMR, Grossberg R, Harkins MS, Mularski RA, Erdmann N, Sandkovsky U, Almasri E, Pineda JR, Dretler AW, de Castilla DL, Branche AR, Park PK, Mehta AK, Short WR, McLellan SLF, Kline S, Iovine NM, El Sahly HM, Doernberg SB, Oh MD, Huprikar N, Hohmann E, Kelley CF, Holodniy M, Kim ES, Sweeney DA, Finberg RW, Grimes KA, Maves RC, Ko ER, Engemann JJ, Taylor BS, Ponce PO, Larson L, Melendez DP, Seibert AM, Rouphael NG, Strebe J, Clark JL, Julian KG, de Leon AP, Cardoso A, de Bono S, Atmar RL, Ganesan A, Ferreira JL, Green M, Makowski M, Bonnett T, Beresnev T, Ghazaryan V, Dempsey W, Nayak SU, Dodd LE, Beigel JH, Kalil AC (2022). Baricitinib versus dexamethasone for adults hospitalised with COVID-19 (ACTT-4): a randomised, double-blind, double placebo-controlled trial. Lancet Respir Med.

[10] Tomazini BM, Maia IS, Cavalcanti AB, Berwanger O, Rosa RG, Veiga VC, Avezum A, Lopes RD, Bueno FR, Silva M, Baldassare FP, Costa ELV, Moura RAB, Honorato MO, Costa AN, Damiani LP, Lisboa T, Kawano-Dourado L, Zampieri FG, Olivato GB, Righy C, Amendola CP, Roepke RML, Freitas DHM, Forte DN, Freitas FGR, Fernandes CCF, Melro LMG, Junior GFS, Morais DC, Zung S, Machado FR, Azevedo LCP (2020). Effect of dexamethasone on days alive and ventilator-free in patients with moderate or severe acute respiratory distress syndrome and COVID-19: the CoDEX randomized clinical trial. JAMA.

[11] Pardo J, Shukla AM, Chamarthi G, Gupte A (2020). The journey of remdesivir: from Ebola to COVID-19. Drugs Context.

[12] Drożdżal S, Rosik J, Lechowicz K, Machaj F, Szostak B, Przybyciński J, Lorzadeh S, Kotfis K, Ghavami S, Łos MJ (2021). An update on drugs with therapeutic potential for SARS-CoV-2 (COVID-19) treatment. Drug Resist Updat.

[13] RECOVERY Collaborative Group (2021). Tocilizumab in patients admitted to hospital with COVID-19 (RECOVERY): a randomised, controlled, open-label, platform trial. Lancet.

[14] Gressens SB, Esnault V, De Castro N, Sellier P, Sene D, Chantelot L, Hervier B, Delaugerre C, Chevret S, Molina JM (2022). Remdesivir in combination with dexamethasone for patients hospitalized with COVID-19: a retrospective multicenter study. PLoS ONE.

[15] COVID-19 RISK and Treatments (CORIST) Collaboration (2020). Use of hydroxychloroquine in hospitalised COVID-19 patients is associated with reduced mortality: findings from the observational multicentre Italian CORIST study. Eur J Intern Med.

[16] Di Castelnuovo A, Costanzo S, Antinori A, Berselli N, Blandi L, Bonaccio M, Bruno R, Cauda R, Gialluisi A, Guaraldi G, Menicanti L, Mennuni M, My I, Parruti A, Patti G, Perlini S, Santilli F, Signorelli C, Stefanini GG, Vergori A, Ageno W, Aiello L, Agostoni P, Al Moghazi S, Arboretti R, Aucella F, Barbieri G, Barchitta M, Bartoloni A, Bologna C, Bonfanti P, Caiano L, Carrozzi L, Cascio A, Castiglione G, Chiarito M, Ciccullo A, Cingolani A, Cipollone F, Colomba C, Colombo C, Crosta F, Dalena G, Dal Pra C, Danzi GB, D'Ardes D, de Gaetano DK, Di Gennaro F, Di Tano G, D'Offizi G, Filippini T, Maria Fusco F, Gaudiosi C, Gentile I, Gini G, Grandone E, Guarnieri G, Lamanna GLF, Larizza G, Leone A, Lio V, Losito AR, Maccagni G, Maitan S, Mancarella S, Manuele R, Mapelli M, Maragna R, Marra L, Maresca G, Marotta C, Mastroianni F, Mazzitelli M, Mengozzi A, Menichetti F, Milic J, Minutolo F, Molena B, Mussinelli R, Mussini C, Musso M, Odone A, Olivieri M, Pasi E, Perroni A, Petri F, Pinchera B, Pivato CA, Poletti V, Ravaglia C, Rossato M, Rossi M, Sabena A, Salinaro F, Sangiovanni V, Sanrocco C, Scorzolini L, Sgariglia R, Simeone PG, Spinicci M, Trecarichi EM, Veronesi G, Vettor R, Vianello A, Vinceti M, Visconti E, Vocciante L, De Caterina R, Iacoviello L, COVID-19 RISK and Treatments (CORIST) Collaboration (2021). Lopinavir/ritonavir and darunavir/cobicistat in hospitalized COVID-19 patients: findings from the multicenter Italian CORIST Study. Front Med.

[17] Zeleke AJ, Moscato S, Miglio R, Chiari L (2022). Length of stay analysis of COVID-19 hospitalizations using a count regression model and quantile regression: a Study in Bologna, Italy. Int J Environ Res Public Health.

[18] Sen-Crowe B, Sutherland M, McKenney M, Elkbuli A (2021). A closer look into global hospital beds capacity and resource shortages during the COVID-19 Pandemic. J Surg Res.

[19] Wang J, Kim S (2021). The paradox of conspiracy theory: the positive impact of beliefs in conspiracy theories on preventive actions and vaccination intentions during the COVID-19 Pandemic. Int J Environ Res Public Health.

[20] Polivka L, Gajdacsi J, Fazekas L, Sebok S, Barczi E, Hidvegi E, Sutto Z, Dinya E, Maurovich-Horvat P, Szabo AJ, Merkely B, Müller V (2022). Long-term survival benefit of male and multimorbid COVID-19 patients with 5-day remdesivir treatment. J Glob Health.

[21] Asselah T, Durantel D, Pasmant E, Lau G, Schinazi RF (2021). COVID-19: discovery, diagnostics and drug development. J Hepatol.

[22] Ichiyama T, Komatsu M, Wada Y, Hanaoka M (2022). Report of a combination of remdesivir, intravenous methylprednisolone pulse, and tocilizumab for severe coronavirus disease: 20-case series at a single institution. Respir Investig.

[23] Kramer A, Prinz C, Fichtner F, Fischer AL, Thieme V, Grundeis F, Spagl M, Seeber C, Piechotta V, Metzendorf MI, Golinski M, Moerer O, Stephani C, Mikolajewska A, Kluge S, Stegemann M, Laudi S, Skoetz N (2022). Janus kinase inhibitors for the treatment of COVID-19. Cochrane Database Syst Rev.

[24] Gavriatopoulou M, Ntanasis-Stathopoulos I, Korompoki E, Fotiou D, Migkou M, Tzanninis IG, Psaltopoulou T, Kastritis E, Terpos E, Dimopoulos MA (2021). Emerging treatment strategies for COVID-19 infection. Clin Exp Med.

[25] Fagbamigbe AF, Tolba MF, Amankwaa EF, Mante PK, Sylverken AA, Zahouli JZB, Goonoo N, Mosi L, Oyebola K, Matoke-Muhia D, de Souza DK, Badu K, Dukhi N (2022). Implications of WHO COVID-19 interim guideline 2020 5 on the comprehensive care for infected persons in Africa Before, during and after clinical management of cases. Sci Afr.

[26] Chhatwal J, Basu A (2022). Cost-effectiveness of remdesivir for COVID-19 treatment: what are we missing?. Value Health.

[27] D'Silva KM, Wallace ZS (2021). COVID-19 and rheumatoid arthritis. Curr Opin Rheumatol.

[28] McMahon JH, Udy A, Peleg AY (2020). Remdesivir for the treatment of Covid-19—preliminary report. N Engl J Med.

[29] Ambrosino I, Barbagelata E, Corbi G, Ciarambino T, Politi C, Moretti AM (2020). Gender differences in treatment of Coronavirus Disease-2019. Monaldi Arch Chest Dis.

[30] Grech V, Savona-Ventura C, Vassallo-Agius P (2002). Research pointers: unexplained differences in sex ratios at birth in Europe and North America. BMJ.

[31] Centers for Disease Control and Prevention, Covid-19 hospitalizations. Centers for Disease Control and Prevention., https://gis.cdc.gov/grasp/COVIDNet/COVID19_5.html (Accessed from 15 Jan 2023).

[32] Senderovich H, Vinoraj D, Stever M, Waicus S (2022). Efficacy of COVID-19 treatments among geriatric patients: a systematic review. Ther Adv Infect Dis.

[33] Procopio G, Cancelliere A, Trecarichi EM, Mazzitelli M, Arrighi E, Perri G, Serapide F, Pelaia C, Lio E, Busceti MT, Pelle MC, Ricchio M, Scaglione V, Davoli C, Fusco P, La Gamba V, Torti C, Pelaia G (2020). Oxygen therapy via high flow nasal cannula in severe respiratory failure caused by Sars-Cov-2 infection: a real-life observational study. Ther Adv Respir Dis.

[34] Vogrig A, Gigli GL, Bnà C (2021). Stroke in patients with COVID19: clinical and neuroimaging characteristics. Neurosci Lett..

[35] Iwamoto S, Johnstone M, Chiu M (2022). Acute ischemic stroke in a young woman with an otherwise asymptomatic SARS-CoV-2 infection. J Med Res Surg.

[36] Di Castelnuovo A, Costanzo S, Antinori A, Berselli N, Blandi L, Bonaccio M, Cauda R, Guaraldi G, Menicanti L, Mennuni M, Parruti G, Patti G, Santilli F, Signorelli C, Vergori A, Abete P, Ageno W, Agodi A, Agostoni P, Aiello L, Al Moghazi S, Arboretti R, Astuto M, Aucella F, Barbieri G, Bartoloni A, Bonfanti P, Cacciatore F, Caiano L, Carrozzi L, Cascio A, Ciccullo A, Cingolani A, Cipollone F, Colomba C, Colombo C, Crosta F, Danzi GB, D'Ardes D, de Gaetano DK, Di Gennaro F, Di Tano G, D'Offizi G, Fantoni M, Fusco FM, Gentile I, Gianfagna F, Grandone E, Graziani E, Grisafi L, Guarnieri G, Larizza G, Leone A, Maccagni G, Madaro F, Maitan S, Mancarella S, Mapelli M, Maragna R, Marcucci R, Maresca G, Marongiu S, Marotta C, Marra L, Mastroianni F, Mazzitelli M, Mengozzi A, Menichetti F, Meschiari M, Milic J, Minutolo F, Molena B, Montineri A, Mussini C, Musso M, Niola D, Odone A, Olivieri M, Palimodde A, Parisi R, Pasi E, Pesavento R, Petri F, Pinchera B, Poletti V, Ravaglia C, Rognoni A, Rossato M, Rossi M, Sangiovanni V, Sanrocco C, Scorzolini L, Sgariglia R, Simeone PG, Taddei E, Torti C, Vettor R, Vianello A, Vinceti M, Virano A, Vocciante L, De Caterina R, Iacoviello L (2021). Heparin in COVID-19 patients is associated with reduced in-hospital mortality: the multicenter Italian CORIST Study. Thromb Haemost.

[37] Stone JH, Frigault MJ, Serling-Boyd NJ, Fernandes AD, Harvey L, Foulkes AS, Horick NK, Healy BC, Shah R, Bensaci AM, Woolley AE, Nikiforow S, Lin N, Sagar M, Schrager H, Huckins DS, Axelrod M, Pincus MD, Fleisher J, Sacks CA, Dougan M, North CM, Halvorsen YD, Thurber TK, Dagher Z, Scherer A, Wallwork RS, Kim AY, Schoenfeld S, Sen P, Neilan TG, Perugino CA, Unizony SH, Collier DS, Matza MA, Yinh JM, Bowman KA, Meyerowitz E, Zafar A, Drobni ZD, Bolster MB, Kohler M, D'Silva KM, Dau J, Lockwood MM, Cubbison C, Weber BN, Mansour MK (2020). Efficacy of tocilizumab in patients hospitalized with Covid-19. N Engl J Med.

[38] Lamb YN (2020). Remdesivir: first approval. Drugs.

[39] COVID-19 Treatment Guidelines Panel. Coronavirus Disease 2019 (COVID-19) Treatment Guidelines. National Institutes of Health. Available at https://www.covid19treatmentguidelines.nih.gov/. Accessed from 17 Jun 2023.

[40] Deana C, Rovida S, Orso D, Bove T, Bassi F, De Monte A, Vetrugno L (2021). Learning from the Italian experience during COVID-19 pandemic waves: be prepared and mind some crucial aspects. Acta Biomed.

[41] Zamani S, Alizadeh M, Shahrestanaki E, Mohammadpoor Nami S, Qorbani M, Aalikhani M, Hassani Gelsefid S, Mohammadian Khonsari N (2022). Prognostic comparison of COVID-19 outpatients and inpatients treated with Remdesivr: a retrospective cohort study. PLoS ONE.

[42] Centers for Disease Control and Prevention, SARS-COV-2 variant classifications and definitions. Centers for Disease Control and Prevention., https://www.cdc.gov/coronavirus/2019-ncov/variants/variant-classifications.html#anchor_1632154493691 Accessed from 15 Jan 2023.

[43] Chavda VP, Bezbaruah R, Deka K, Nongrang L, Kalita T (2022). The Delta and Omicron variants of SARS-CoV-2: what we know so far. Vaccines.

[44] Muhar BK, Chu H, Zhou N (2022). Retrospective cross-sectional analysis of COVID-19 patients in a local hospital during delta surge. J Health Care Res.

